# Assessing Alcohol Dependence in Hospitalized Patients

**DOI:** 10.3390/ijerph110605783

**Published:** 2014-05-28

**Authors:** Juliana Doering-Silveira, Thiago Marques Fidalgo, Carolina Lins E. Silva Nascimento, Juliana Bernardo Alves, Caroline Lumy Seito, Maria Claudia Saita, Lorenza Oliveira Belluzzi, Laila Carolina Silva, Dartiu Silveira, Leonardo Rosa-Oliveira

**Affiliations:** Department of Psychiatry, Federal University of São Paulo, São Paulo School of Medicine, Rua Prof. Ascendino Reis 763, Sao Paulo, Brazil; E-Mails: Doering-Silveira@unifesp.br (J.D.-S.); Nascimento@unifesp.br (C.L.E.S.N.); alves@unifesp.br (J.B.A.); seito@unifesp.br (C.L.S.); saita@unifesp.br (M.C.S.); Belluzzi@unifesp.brv (L.O.B.); silva@unifesp.br (L.C.S.); dartiu@terra.com.br (D.S.); oliveira@unifesp.br (L.R.-O.)

**Keywords:** alcohol, assessment, general hospital

## Abstract

Alcohol misuse is generally not detected in hospital settings. The goal of this study was to estimate the prevalence of alcohol abuse and dependence in hospitalized patients in a university hospital in Sao Paulo (Brazil). Patients were randomly selected from all hospital admissions. The final sample consisted of 169 adult inpatients. Two screening tools were used: the Short Alcohol Dependence Data (SADD) and the CAGE questionnaires. In this sample, 25.4% of patients could be considered alcohol dependent according to the CAGE questionnaire, whereas 32.9% of patients fulfilled the criteria according to the SADD. The only predictor of alcohol dependence was gender; male inpatients were 3.2 times more prone to alcohol dependence with female inpatients. All inpatients should be systematically screened for alcohol use disorders. The choice of the screening tool will depend on whether the goal is to identify inpatients with hazardous drinking behaviors or with established alcohol-related problems. To maximize proper case identification, the CAGE questionnaire should be used as a first-step screening tool, and patients who screen positive on this scale should be subsequently administered the SADD questionnaire to assess the severity of the condition.

## 1. Introduction

Alcohol abuse and dependence, as defined by DSM-IV criteria, are considered severe medical conditions that have assumed enormous dimensions, particularly over the last 50 years [1]. Furthermore, the burden of disability and morbidity related to alcohol misuse is well established worldwide [2].

Affecting virtually every bodily system, alcohol has been implicated not only in liver disease, but also in hypertension, myocardial disorders, immune dysfunction, neurological and psychiatric disorders, and other conditions. Studies involving hospitalized patients indicate that up to one-third of patients admitted to medical and surgical wards have alcohol-related conditions [3]. Huang *et al.* have found a life time prevalence of alcohol dependence among mental illness inpatients of 8.3%, whereas the prevalence of alcohol abuse and alcohol use disorders was of 1.5% and 9.8%, respectively [4]. A study conducted in Taiwan found a prevalence of alcohol use disorder in the previous year of 25.7% among inpatients [5]. This study added significant information. Only 14.1% of nonpsyhiatric physicians detected patients with recent alcohol use disorders [5]. Chen *et al.* had also found a low detection rate of alcohol related-problems by clinicians. This study found a prevalence of alcohol abuse and alcohol dependence of 3.9% and 12.6%, respectively, in the previous year. The authors conclude that approximately one sixth of nonpsychiatric inpatients in a general hospital have alcohol use disorders and have been neglected by medical staff [6]. In summary, alcohol use disorders are high prevalent conditions, but often not detected in hospital settings, despite the availability of brief screening scales [7,8,9].

The main objective of the present investigation was to estimate the prevalence of alcohol abuse and dependence in hospitalized patients in a university hospital. Moreover, we aimed to evaluate two screening instruments performance among this population. 

## 2. Methods

The study was conducted in Sao Paulo Hospital, Brazil, from April to June 2007.

### 2.1. Sample

All wards were included in the study, with the exception of the ICU unit, the Pediatric unit and the Psychiatric unit. This approach resulted in 495 patients for potential inclusion in the study. All patients who did not fulfill the exclusion criterion were invited to participate in the study. 

The exclusion criterion included the presence of a general condition that did not permit the patient to respond to a structured questionnaire under the guidance of a medical student. These conditions included, for example, delirium, dementia or any type of limitation, such as deafness. 

### 2.2. Instruments and Procedures

Two screening tools were used: the Short Alcohol Dependence Data (SADD) [10] and the CAGE [11] questionnaires. 

The SADD questionnaire was used to measure the severity of alcohol dependence [12]. Based on the Edwards and Gross formulation of alcohol dependence syndrome [13], this 15-item self-report questionnaire provides a measure of the severity of alcohol dependence on a *continuum*, which ranges from a mild drinking problem to severe alcohol dependence. The following scores are used to quantify the severity of alcohol dependence: no dependence, score equal to zero; mild dependence, score greater than zero and less than or equal to 9; moderate dependence, score greater than 9 and less than or equal to 19; and severe dependence, score ranging from 20 to 45.

The CAGE is a four-question screening scale used to identify individuals at risk of alcohol abuse or dependence. Positive answers to two or more questions indicate probable alcohol abuse [11]. In terms of its psychometric properties, sensitivities of more than 80%, specificities greater than 85%, and positive predictive values of up to 82% have been reported [14,15,16]. Although the psychometric properties of the CAGE questionnaire have previously been an issue of discussion, its use among inpatients was well established [17]. We have used the Brazilian version, validated by Masur [18]. In this study, the instrument presented sensibility of 0.88 and specificity of 0.83. Castels, in a study with 747 Brazilian inpatients, found sensibility of 0.94 and specificity of 0.85 [19]. Thus, this seems to be a good instrument to be used among inpatients, especially in the Brazilian context.

The reason we have used these two instruments is because they are widely studied instruments, simple of being used. Moreover, they are fast, reliable tools, which can be used by any health professional, requiring low level of training. ICD or DSM criteria were not used due to its complexity and due to the fact that they are time consuming tools, which require extensive training. 

### 2.3. Ethical Issues

The patients were interviewed by medical students from the Federal University of Sao Paulo, Brazil. The patients answered the questionnaires during hospitalization, at which time they were told the objectives of the investigation. The participants were asked to sign an informed consent form according to the standards of the Research Ethics Committee of the Federal University of Sao Paulo.

### 2.4. Data Analysis

The variables were tested in order to check if they had a normal distribution, which was confirmed. Chi-square tests were used to analyze categorical data, whereas t-tests were used to analyze parametric continuous variables. Logistical regression was used to examine the interrelationships between multiple variables. Differences between groups were considered significant at the *p* < 0.05 level.

## 3. Results

**Figure 1:**
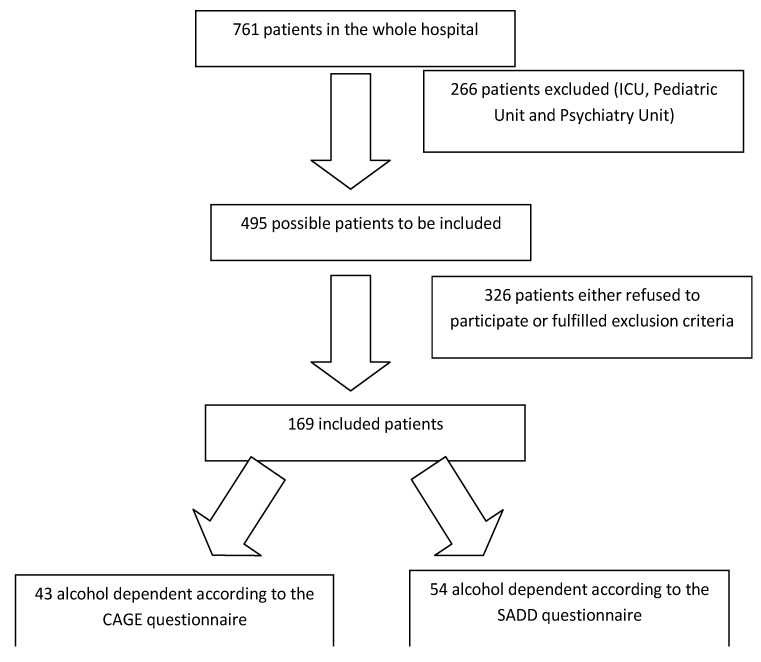
Flow chart describing patient participation.

The final sample consisted of 169 inpatients (34.1% of the 495 possible patients) in a university hospital of the Federal University of Sao Paulo, Brazil. A flow chart describing patient participation can be seen on Figure 1. No data are available concerning patients not included in the study. Eighty-three (49.1%) patients were male and 86 (50.9%) patients were female, with a mean age of 47.7 ± 15.6 years. The subjects were predominantly Caucasian (65.1%), married (54.4%), and unemployed (64.5%), and most participants (88.7%) had completed at least elementary school. The high rate of unemployment may be because the hospital studied is a public hospital. In Brazil, individuals with a low income often present for treatment at these facilities. 

In this study, 43 (25.4%) patients were considered with a high probability of presenting an alcohol use disorder according to the CAGE questionnaire (Table 1), whereas 54 (32.9%) patients fulfilled the criteria for alcohol dependence according to the SADD questionnaire (Table 2), with a mean score of 7.96 ± 6.8. In the entire sample, 115 (68.0%) patients scored zero on the SADD questionnaire, whereas 39 (23.1%) patients had a low level of alcohol dependence (SADD ranging from 1 to 9). In addition, 10 (5.9%) patients had a medium level of dependence (SADD ranging from 10 to 19), and only 5 (3.0%) patients had a high level of alcohol dependence (SADD ranging from 20 to 45).

The mean age of initial alcohol use in alcohol dependent patients was 14.9 ± 3.8 years, whereas the mean age of initial alcohol use in non-dependent patients was 15.4 ± 6.8 years. The number of years that alcohol dependent individuals had used alcohol was 29.4 ± 18.0 years, whereas non-dependent individuals had used alcohol for 33.6 ± 18.9 years. These differences did not reach statistical significance (Table 3).

Fifteen (25.9%) clinical ward patients were alcohol dependent, and 25 (28.7%) surgical patients were considered alcohol dependent. In addition, only 3 (12.5%) gynecologic/obstetric patients were alcohol dependent.

A logistical regression was performed to evaluate the influence of multiple variables on predicting alcohol dependence. Socio-demographic variables were tested (gender, age, work status, marital status, educational background) as well as the diagnosis (problematic or non-problematic alcohol use) and the initial age of alcohol use (total of seven variables). The only predictor found was gender; male inpatients were 3.2 times more prone to being considered alcohol dependent compared with female inpatients (*p* < 0.01; CI: 1.51–6.67). It is important to emphasize that all women were analyzed as a unique group, whether they were admitted in a general ward or in the gynecologic/obstetric ward.

**Table 1:** CAGE results.

CAGE Results	
CAGE positive	43 (25.4%)
CAGE negative	126 (74.6%)

**Table 2:** SADD results.

SADD Results	
no dependence	115 (68.0%)
low level of alcohol dependence (score 1 to 9)	39 (23.1%)
medium level of alcohol dependence (score 10 to 19)	10 (5.9%)
high level of alcohol dependence (score 20 to 45)	5 (3.0%)
Total	169 (100%)

**Table 3:** Comparison between alcohol dependents and non-dependent patients.

	SADD Results		
	Alcohol Dependents	Alcohol Non-Dependents	Statistics
Initial age in years of alcohol use	14.9 ± 3.8	15.4 ± 6.8	*p* > 0.05
Number of years using alcohol	29.4 ± 18.0	33.6 ± 18.9	*p* > 0.05

## 4. Discussion

Our findings are consistent with the results of other studies on the prevalence of alcohol-related disorders in hospitalized patients [14,20]. In our study, prevalence rates of alcohol use disorders were of 25.4% and of 32.9%, depending of the screening instrument used. These numbers are similar to a Taiwan study [5], which found a prevalence rate of 25.7%. Another study [6] found an alcohol dependence prevalence of 3.9%, which is comparable to the high alcohol dependence rate found in our study using the SADD, of 3.0%. Lower rates were found by Huang *et al.* [4]. However, this study investigates patients with severe mental illness, which may bias comparisons.

The CAGE questionnaire is a four-item screening tool that can be easily administered in hospital settings. The CAGE questionnaire provides good case identification for alcohol abuse and dependence, with false-positive rates of approximately 5% [18]; other screening tools, such as the AUDIT, may reach false-positive rates of up to 30% [21]. Moreover, to our knowledge, there is only one validation to Brazilian context study concerning AUDIT and it was made among river population in Brazilian Amazonas. Among young men, the specificity of the AUDIT (compared with a DSM-IV diagnosis) may be less than 47% [22]. In the present study, the CAGE questionnaire identified 25.4% of inpatients as having a probable diagnosis of alcohol abuse or dependency. 

Because the SADD questionnaire is considered to have a high specificity for alcohol dependence syndrome but a low sensitivity, making it unsuitable as a screening tool for general inpatients, we consider the CAGE questionnaire results as more reliable because they are a better estimate of alcohol dependence. Alternatively, the SADD questionnaire allowed us to estimate the severity of the condition. Saitz *et al.* [23] found that 81% of patients who were positive on screening instruments for alcohol problems were confirmed to be alcohol dependent during a detailed interview. 

It is interesting to note that Henkel *et al.* [24] found that unemployment had a strong connection to drinking problems in men. In our study, we found that being a man was a risk factor for presenting with a drinking problem. Although none of the socio-demographic variables were included in the final logistic regression model, our sample had a high rate of unemployed subjects.

Alcohol comorbidity continues to be neglected in the assessment and treatment of other medical conditions [25,26,27]. Patients with alcohol-related problems are seldom identified or referred. Physicians may underestimate the importance of alcohol as a comorbid risk factor and are generally unaware of the benefits of early interventions. Furthermore, they are often not acquainted with the efficiency of existing screening tools and are also uncertain about the tools’ ability to accurately identify alcohol-related problems. 

In summary, all inpatients should be systematically screened for alcohol use disorders. The choice of the screening tool will depend on whether the goal is to identify all inpatients with hazardous drinking behavior or only inpatients with established alcohol-related problems. To maximize proper case-identification, the CAGE questionnaire should be used as a first step screening tool, and patients with a positive screen on this scale should be subsequently administered the SADD questionnaire for severity assessment. It is important to state that all patients identified as positive on the CAGE should be submitted to a diagnosis interview. This, however, may not be possible, due to lack of physicians or due to the high number of patients. This is especially true in low or middle income countries. In this context, counting on an instrument such as SADD, which could help on determining those patients who should be submitted to a diagnosis interview first may be useful.

## 5. Limitations

Some limitations of this study must be considered. Although all patients of the chosen wards were invited to participate, only one hospital was included, thus, results may not be extrapolated. Although it is a tertiary hospital in a large city, this environment may have biased the results. Moreover, patients with severe conditions, such as delirium or dementia, were not included in the study. Severe alcohol dementia may lead to alcoholic dementia and in this case we might have lost some patients that would be screened positively. Moreover, alcohol withdrawal is a condition that can lead to the development of delirium. Once again, more severe dependents may have been lost due to this exclusion criteria. High refusal rates may also be a bias and may be due to the fact that the research was conducted in a university hospital, where many research protocols are conducted. Therefore, patients may get tired of answering many questionnaires and of being submitted to repeated interviews by researchers and by residents and students who work at the facility. 

Finally, no gold standard was chosen for defining alcohol dependence. Thus, our study may have detected a significant group of false positives.

## 6. Conclusions

All inpatients should be systematically screened for alcohol use disorders, in order to minimize under diagnoses of these conditions, as they may influence patients’ treatment and prognosis. The choice of the screening tool will depend on whether the goal is to identify inpatients with hazardous drinking behavior or with established alcohol-related problems. To maximize proper case identification, the CAGE questionnaire should be used as a first-step screening tool, and patients who screen positive on this scale should be subsequently administered the SADD questionnaire for severity assessment.

## References

[1] World Health Organization (WHO) (1986). Problems Related to Alcohol Consumption.

[2] World Health Organization (WHO) (1999). Global Status Report on Alcohol.

[3] UK Alcohol Forum (1997). Guidelines for the Management of Alcohol Problems in Primary Care and General Psychiatry.

[4] Huang M.C., Yu C.H., Chen C.T., Chen C.C., Shen W.W., Chen C.H. (2009). Prevalence and identification of alcohol use disorders among severe mental illness inpatients in Taiwan. Psychiatry Clin. Neurosci..

[5] Wu S.I., Liu S.I., Fang C.K., Hsu C.C., Sun Y.W. (2006). Prevalence and detection of alcohol use disorders among general hospital inpatients in eastern Taiwan. Gen. Hosp. Psychiatry.

[6] Chen C.H., Chen W.J., Cheng A.T. (2004). Prevalence and identification of alcohol use disorders among nonpsychiatric inpatients in one general hospital. Gen. Hosp. Psychiatry.

[7] World Health Organization Brief Interview Study Group (1996). A cross-national trial of brief intervention with heavy drinkers. Am. J. Publ. Health.

[8] Bien T.H., Miller W.R., Tonigan J.S. (1993). Brief interventions for alcohol problems: A review. Addiction.

[9] Wallace P., Cutler S., Haines A. (1988). Randomised controlled trial of general practitioner intervention in patients with excessive alcohol consumption. BMJ.

[10] Saunders J.B., Aasland O.G., Amundsen A., Grant M. (1993). Alcohol consumption and related problems among primary health care patients: Who collaborative project on early detection of persons with harmful alcohol consumption—I. Addiction.

[11] Ewing J.A. (1984). Detecting alcoholism. The cage questionnaire. JAMA.

[12] Raistrick D., Dunbar G., Davidson R. (1983). Development of a questionnaire to measure alcohol dependence. Br. J. Addict..

[13] Davidson R., Raistrick D. (1986). The validity of the Short Alcohol Dependence Data (SADD) Questionnaire: A short self-report questionnaire for the assessment of alcohol dependence. Br. J. Addict..

[14] Soderstrom C.A., Smith G.S., Kufera J.A., Dischinger P.C., Hebel J.R., McDuff D.R., Gorelick D.A., Ho S.M., Kerns T.J., Read K.M. (1997). The accuracy of the cage, the brief Michigan alcoholism screening test, and the alcohol use disorders identification test in screening trauma center patients for alcoholism. J. Trauma.

[15] Mackenzie D., Langa A., Brown T.M. (1996). Identifying hazardous or harmful alcohol use in medical admissions: A comparison of audit, cage and brief mast. Alcohol Alcoholism.

[16] Chan A.W., Pristach E.A., Welte J.W. (1994). Detection by the cage of alcoholism or heavy drinking in primary care outpatients and the general population. J. Substance Abuse.

[17] Aertgeerts B., Buntinx F., Kester A. (2004). The value of the CAGE in screening for alcohol abuse and alcohol dependence in general clinical populations: A diagnostic meta-analysis. J. Clin. Epidemiol..

[18] Masur J., Monteiro M.G. (1983). Validation of the “CAGE” alcoholism screening test in a Brazilian psychiatric inpatient hospital setting. Braz. J. Med Biol. Res..

[19] Castells M.A., Furlanetto L.M. (2005). Validity of the CAGE questionnaire for screening alcohol-dependent inpatients on hospital wards. Rev. Bras. Psiquiatr..

[20] Schnuerer I., Gaertner B., Baumann S., Rumpf H.J., John U., Hapke U., Freyer-Adam J. (2013). Gender-specific predictors of risky alcohol use among general hospital inpatients. Gen. Hosp. Psychiatry.

[21] Moretti-Pires R.O., Corradi-Webster C.M. (2011). Adaptation and validation of the Alcohol Use Disorders Identification Test (AUDIT) for a river population in the Brazilian Amazon. Cad. Saude. Publica..

[22] Hearne R., Connolly A., Sheehan J. (2002). Alcohol abuse: Prevalence and detection in a general hospital. J. R. Soc. Med..

[23] Saitz R., Freedner N., Palfai T.P., Horton N.J., Samet J.H. (2006). The severity of unhealthy alcohol use in hospitalized medical patients. The spectrum is narrow. J. Gen. Intern. Med..

[24] Henkel D. (2011). Unemployment and substance use: A review of the literature (1990–2010). Curr. Drug Abuse Rev..

[25] Henni A., Bideau C., Routon X., Berrut G., Cholet J. (2013). Prevalence and issues of screening for alcohol consumption among elderly inpatients admitted to acute geriatric inpatient unit. Geriatr. Psychol. Neuropsychiatr. Vieil..

[26] Saitz R., Palfai T.P., Cheng D.M., Horton N.J., Dukes K., Kraemer K.L., Roberts M.S., Guerriero R.T., Samet J.H. (2009). Some medical inpatients with unhealthy alcohol use may benefit from brief intervention. J. Stud. Alcohol Drugs.

[27] Saitz R., Palfai T.P., Cheng D.M., Horton N.J., Freedner N., Dukes K., Kraemer K.L., Roberts M.S., Guerriero R.T., Samet J.H. (2007). Brief intervention for medical inpatients with unhealthy alcohol use: A randomized, controlled trial. Ann. Intern. Med..

